# How is tailored implementation undertaken using a self-guided toolkit? Qualitative study of the ItFits-toolkit in the ImpleMentAll project

**DOI:** 10.1186/s13012-024-01380-w

**Published:** 2024-07-11

**Authors:** Tracy L. Finch, Sebastian Potthoff, Carl R. May, Melissa Girling, Neil Perkins, Christiaan Vis, Leah Bührmann, Anne Etzelmueller, Claire Rosalie van Genugten, Josien Schuurmans, Jordi Piera-Jiménez, Tim Rapley, on behalf of the ImpleMentAll consortium, Adriaan Hoogendoorn, Ainslie O’Connor, Alexis Whitton, Alison Calear, Andia Meksi, Anna Sofie Rømer, Anne Etzelmüller, Antoine Yrondi, Arlinda Cerga-Pashoja, Besnik Loshaj, Bridianne O’Dea, Bruno Aouizerate, Camilla Stryhn, Carmen Ceinos, Caroline Oehler, Catherine Pope, Christine Marking, Claus Duedal Pedersen, Corinna Gumbmann, Dana Menist, David Daniel Ebert, Denise Hanssen, Elena Heber, Els Dozeman, Emilie Brysting, Emmanuel Haffen, Enrico Zanalda, Erida Nelaj, Erik Van der Eycken, Eva Fris, Fiona Shand, Gentiana Qirjako, Géraldine Visentin, Heleen Riper, Helen Christensen, Ingrid Titzler, Isabel Weber, Isabel Zbukvic, Jeroen Ruwaard, Jerome Holtzmann, Johanna Freund, Johannes H. Smit, Josep Penya, Josephine Kreutzer, Judith Rosmalen, Juliane Hug, Kim Mathiasen, Kristian Kidholm, Kristine Tarp, Linda Lisberg, Ludovic Samalin, Maite Arrillaga, Margot Fleuren, Maria Chovet, Marion Leboyer, Mette Atipei Craggs, Mette Maria Skjøth, Naim Fanaj, Nicole Cockayne, Philip J. Batterham, Pia Driessen, Pierre Michel Llorca, Rhonda Wilson, Ricardo Araya, Robin Kok, Sergi García Redondo, Sevim Mustafa, Søren Lange Nielsen, Ulrich Hegerl, Virginie Tsilibaris, Wissam Elhage, Ylenia Sacco

**Affiliations:** 1 Department of Nursing, Midwifery & Health, Northumbria University, Newcastle Upon Tyne, NE7 7XA UK; 2 Department of Social Work, Education & Community Wellbeing, Northumbria University, Newcastle Upon Tyne, NE7 7XA UK; 3 Faculty of Public Health and Policy, London School of Hygiene and Tropical Medicine, London, WC1H 9SH UK; 4 Department of Public and Occupational Health, Amsterdam Public Health Research Institute, Amsterdam UMC, Amsterdam, the Netherlands; 5 HelloBetter/GET.ON Institute, 20249 Hamburg, Germany; 6 Clinical, Neuro-, & Developmental Psychology, Faculty of Behavioural and Movement Sciences, Vrije Universiteit Amsterdam, Amsterdam, NL the Netherlands; 7 Amsterdam Public Health Research Institute, Amsterdam, NL the Netherlands; 8 Focus Therapy, Amsterdam, Netherlands; 9 Catalan Health Service, Barcelona, Spain; 10 Digitalization for the Sustainability of the Healthcare System DS3-IDIBELL, L’Hospitalet de Llobregat, Spain; 11 Faculty of Informatics, Multimedia and Telecommunications, Universitat Oberta de Catalunya, Barcelona, Spain

**Keywords:** Implementation strategies, Tailoring, Toolkit, Implementers, Self-guidance, Determinants assessment

## Abstract

**Background:**

The process of tailored implementation is ill-defined and under-explored. The ItFits-toolkit was developed and subsequently tested as a self-guided online platform to facilitate implementation of tailored strategies for internet-based cognitive behavioural therapy (iCBT) services. In ImpleMentAll, ItFits-toolkit had a small but positive effect on the primary outcome of iCBT normalisation. This paper investigates, from a qualitative perspective, how implementation teams developed and undertook tailored implementation using the toolkit within the trial.

**Methods:**

Implementation teams in thirteen sites from nine countries (Europe and Australia) used the ItFits-toolkit for six months minimum, consistent with the trial protocol. A qualitative process evaluation was conducted. Descriptive data regarding goals, barriers, strategies, and implementation plans collected within the toolkit informed qualitative data collection in real time. Qualitative data included remote longitudinal interviews (*n* = 55) with implementation team members (*n* = 30) and observations of support calls (*n* = 19) with study sites. Qualitative data were analysed thematically, using a team-based approach.

**Results:**

Implementation teams developed and executed tailored implementation projects across all steps in the toolkit process. Working in a structured way but with room for flexibility, decisions were shaped by team members’ ideas and goals, iterative stakeholder engagement, internal and external influences, and the context of the ImpleMentAll project. Although teams reported some positive impacts of their projects, ‘time’, both for undertaking the work, and for seeing project impacts, was described as a key factor in decisions about implementation strategies and assessments of success.

**Conclusion:**

This study responds directly to McHugh et al.’s (2022) call for empirical description of what implementation tailoring looks like in action, in service settings. Self-guided facilitation of tailored implementation enables implementers in service settings to undertake tailoring within their organisations. Implementation tailoring takes considerable time and involves detailed work but can be supported through the provision of implementation science informed guidance and materials, iterative and ongoing stakeholder engagement, and working reflectively in response to external influencing factors. Directions for advancement of tailored implementation are suggested.

**Supplementary Information:**

The online version contains supplementary material available at 10.1186/s13012-024-01380-w.

Contributions to the literature

- Demonstrates how ItFits-toolkit, as an approach that translates implementation science approaches into a user-focused structured self-guided process, can support tailored implementation work in practice settings
- Understanding of how implementation teams practically enact tailored implementation in practice is advanced, through rich description of implementers’ work
- Conceptualisation of tailored implementation is advanced, by prioritising ongoing stakeholder engagement, and time and space to work flexibly and with reflexivity, within a structured process

## Introduction

Implementation science (IS) has developed out of a growing recognition that evidence-based services and interventions are necessary but not sufficient conditions for improving care. Implementation success should be enhanced by identifying, developing, and applying effective implementation strategies [1, 2]. The Expert Recommendations for Implementing Change (ERIC) taxonomy developed by Powell and colleagues [2] includes 73 discrete implementation strategies, that can serve as a ‘menu’ of potential strategies that may be relevant to specific implementation projects. Such implementation strategies are likely to be successful to the extent that they are ‘well matched’ to known or anticipated barriers and facilitators to the implementation of the target intervention or service [1]. Within the field of IS, this process has been described as tailored implementation or implementation tailoring.

Whilst implementation tailoring has been defined in a number of ways [3–5], at its core are two inter-related elements: (1) the identification of determinants of practice, and (2) the selection of strategies to address those determinants of practice. Albers et al. [6] describes tailored implementation as a *prospective process*:

‘Tailored implementation is a prospective process involving the (1) identification and prioritization of barriers and/or facilitators (i.e., determinants) likely to influence the implementation of RSIs (research supported interventions) and (2) selection, operationalization, and application of implementation strategies likely to address the identified determinants’ ([6] p.1).

In this paper we specifically focus on implementation tailoring, as opposed to ‘intervention tailoring’. The latter focuses on tailoring the research supported intervention based on specific characteristics and needs of an individual person (e.g. patients) [7, 8]. Regarding tailored implementation, the scientific evidence is not conclusive when comparing tailored to non-tailored implementation, with studies reporting small to moderate effectiveness [9] to non-effectiveness [10, 11].

Powell et al. [12] and McHugh et al. argue [13] that the concept of implementation tailoring itself remains ill-defined. To advance tailoring as a key process in IS, they identify several key areas of investigation that include exploring what constitutes tailoring as a process, how it works (or is expected to), whom and what activities are involved, enhanced methods for designing and tailoring implementation strategies, and appropriate methods of evaluating their impact [14]. Greater transparency in methodology and clearer reporting – both in relation to tailoring [13] and implementation strategies more specifically [12] is needed.

In this study, we aimed to advance understanding of the process of tailored implementation by examining how implementation teams develop tailored implementation strategies when using a self-guided toolkit designed to facilitate this activity. The Integrated Theory-based Framework for Intervention Tailoring Strategies (the ItFits-toolkit) was developed as part of the ImpleMentAll (IMA) project [15], which focused on improving the implementation of internet-based Cognitive Behavioural Therapy (iCBT) in routine care. In iCBT, therapies for psychological intervention in a wide range of common mental health conditions are grounded in traditional cognitive behavioural therapy practice but provided with the support of internet-based technology to expand opportunity for level and mode of access to services. The effectiveness of iCBT as a treatment is established [16] so the problem of focus here is not on developing or tailoring these iCBT interventions. The objective is to facilitate improved implementation: delivery and organisation of iCBT into mental health services that have been historically provided face-to-face, and where the factors influencing successful integration into routine practice are likely diverse and context-specific [17].

**Fig. 1:**
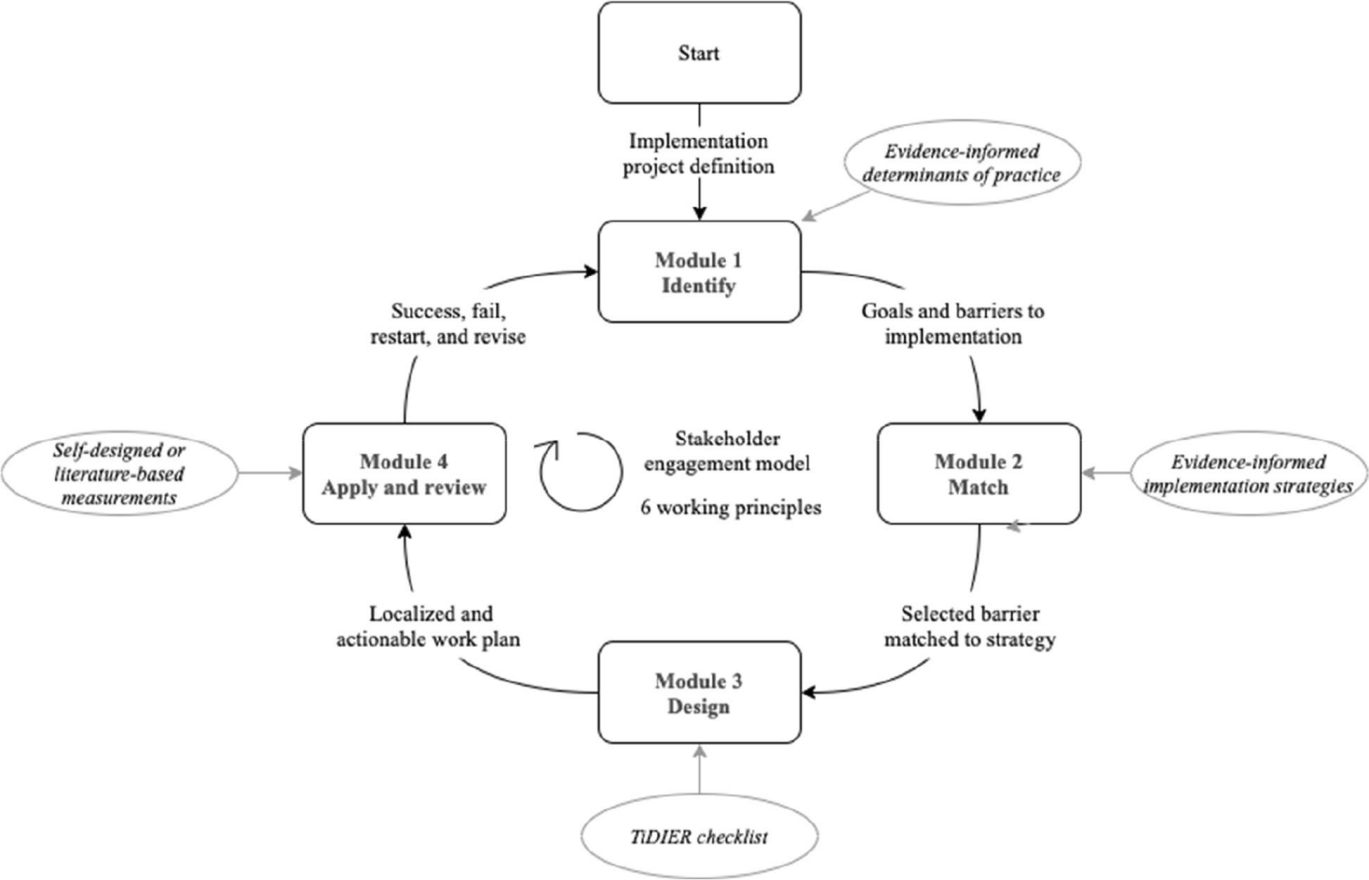
ItFits-toolkit process

ItFits-toolkit is an evidence- and theory-informed [18] self-guided process that provides implementers with guidance, resources, and support to identify and address key barriers to iCBT implementation, using tailored implementation strategies (see Fig. 1), whilst allowing local implementers to drive the tailoring process. The ItFits-toolkit is designed to support ‘implementation tailoring’, not ‘intervention tailoring’, as indicated in the toolkit title (which is to be reconsidered but retained here in original form for consistency with project publications). Accessed via an interactive open access digital platform [19], it enables local teams of implementers to work closely with service delivery staff and other relevant stakeholders to design and apply implementation strategies.

In the ImpleMentAll study, ItFits-toolkit was evaluated in a randomised stepped wedge trial, for its effectiveness as a tool for achieving service implementation related outcomes of iCBT interventions, focusing on level of normalisation (embedding and integration into service provision (primary outcome) [20]) and service uptake (secondary outcome) of the iCBT services into routine healthcare, during the trial [15]. The results showed a significant, but small, positive effect of ItFits-toolkit on the primary outcome of the normalisation of iCBT services in mental health professionals, and no significant effect on service uptake and referrals on patient level [17]. To understand engagement with ItFits-toolkit as a tailoring intervention, we conducted a qualitative process evaluation [15] alongside the trial effectiveness study [17], to understand the ways in which implementers in the IMA study sites worked with the ItFits-toolkit, and the projects that they developed to enhance iCBT delivery.

In this paper, we draw on qualitative process evaluation data to explore the question of: how did implementation teams develop and undertake tailored implementation using the toolkit within the trial?

## Methods

We describe this study using the Standards for Reporting Implementation Studies (StaRI) [21] (see Additional file 1).

### The It-Fits toolkit process

After an initial set-up, the toolkit consists of four substantive modules (see Fig. 1) that guide users through steps of an implementation process, drawing on approaches and tools from IS. Work with and around the toolkit is based on five core mechanisms (see Table 1), with six guiding principles:

**Table 1:** ItFits-toolkit mechanisms and guiding principles

Mechanisms and principles	
M1. Non-standardised, systematically guided step-by-step process	
M2. Stakeholder-based co-creation of solutions	
M3. Tools to identify local barriers, consult stakeholders, and match to suitable strategies	
M4. Evidence-informed materials on barriers, strategies, and intervention planning	
M6. Six guiding principles	P1: ***Be pragmatic***—Focus on realistic, achievable, next steps
	P2: ***Be focused***—Focus on one thing at a time, don't try to do everything at once
	P3: ***Be different***—Do not only focus on things that you feel most comfortable with or the things you would normally do
	P4: ***Be open***—Listen to and value your stakeholders’ knowledge and experience
	P5: ***Be organised***—Each step needs an identified owner to take responsibility for delivery
	P6: ***Be flexible***—The same solution may not work for everyone, so be prepared to adapt your plans and ideas

Module 1 involves identifying and prioritising implementation goals and barriers to reaching these goals, drawing on current systematic review evidence about determinants of iCBT implementation [22]. This taxonomy presents 37 determinants (or ‘factors’ influencing iCBT implementation) categorised into: acceptance, appropriateness, engagement, resources, work processes, and leadership. Module 2 involves matching up implementation barriers to strategies, using the ERIC taxonomy [2], combined with Acceptability, Practicability, Effectiveness, Affordability, Side-effects, and Equity (APPEASE) criteria [23] for strategy assessment. Consistent with the toolkit principles (Table 1), implementers were asked to work towards prioritising one goal, barrier and category of strategy for focus within individual projects, but multiple projects could be developed simultaneously to intentionally allow flexibility in complex settings. Module 3 requires implementers to design a plan for carrying out strategies in a local context, using the Template for Intervention Description and Replication (TIDieR) framework for intervention reporting [24] to detail implementation plans and specify success criteria for later assessment. In Module 4, implementers apply strategies, reviewing progress and adapt as needed. Here, implementers can decide from the following options: ‘stop as success’ (strategy judged to have addressed the barrier), ‘continue’ (with current barrier and strategy), or to go back to a different point in the toolkit to focus on a different goal, barrier, or strategy. The toolkit was presented in English language.

### Deployment of ItFits-toolkit in the trial

The ItFits-toolkit was rolled out within the IMA trial, as part of a stepped-wedge study design with randomised entry into the trial phase [15, 17]. Between December 2018 and March 2020, two sites crossed-over to the ItFits-toolkit condition every three months, until 12 sites were receiving the ItFits-toolkit.

In each trial site, an implementation core team (up to 4 team members and an Implementation Lead) was established. Sites were asked to work with the toolkit for at least six months, aiming to finish a complete cycle of the ItFits-toolkit process (all four modules) within this time to achieve an adequate exposure to the core working components of the toolkit. Teams were advised that they could create multiple projects if they wished. Training on using the toolkit was provided at each site. Periodic support, including monthly teleconferences with sites during their active phase, was provided. Support focused on technical questions about the toolkit, but not on implementation issues as the latter constituted the self-guided work that implementation teams were expected to undertake independently.

Reported previously [17], engagement with the toolkit within the trial was adequate. As, a total of 31 projects were initiated, ranging between 1 and 6 per site. For 12 of the projects, sites completed a full cycle of the tailored implementation process. Eight of the projects were completed up to Module 3, indicating a designed strategy was being implemented. Ten out of the 13 sites progressed at least one project to Module 4, thus considered to have had adequate ‘exposure’ [17]. The projects developed by the implementation teams are summarised in Table 2.

**Table 2:** Descriptive summary of projects developed using ItFits-toolkit

Module 1 – Identify goals and barriers				Module 2 – Match strategies*	Module 4 – Assess plans	ItFits completion
**Target groups**	**End goal**	**Goal emphasis**	**Barriers**			
iCBT service delivery personnel (*n* = 25) Patients & families (*n* = 3) Both (*n* = 3)	Increasing uptake to the iCBT service (*n* = 30) Efficiency e.g., improving the suitability of patients being referred into the service (*n* = 1)	Increasing referral numbers (*n* = 9) Increasing therapy numbers (*n* = 9) Increasing engagement (*n* = 10) Raising awareness of the service (*n* = 2) Increasing referral suitability (*n* = 1)	Acceptance (*n* = 15) Appropriateness (*n* = 1) Engagement (*n* = 2) Resources (*n* = 0) Working Processes (*n* = 3) Leadership (*n* = 0) Healthcare System (*n* = 3) Custom (*n* = 7)	Educate (*n* = 16) Plan (*n* = 5) Quality Management (*n* = 3) Finance (*n* = 0) Restructure (*n* = 1) Policy Context (*n* = 0) Custom (*n* = 4) *2 projects did not reach this stage	Using referral numbers or surveys (*n* = 16) Using qualitative approach e.g., interviews (*n* = 1)	Fully completed (up to module 4) (*n* = 12) Up to module 3 (*n* = 8)

*n* number of projects registered in the toolkit

*Custom barriers* stated by toolkit users as: request form; technological; lack of strategies and priorities (*n* = 3); perceived inconvenience; perceived lack of ownership

*Custom strategies* stated by toolkit users as: ‘automatic’ (automating a process in the system); facilitate relay of clinical data to providers; assess for readiness and identify barriers; seeking advice from other iCBT providers

### Study design

This paper draws on the qualitative data from the process evaluation, that focused on implementation teams’ engagement with the toolkit to develop implementation projects. A completed Standards for Reporting Qualitative Research (SRQR) [25] checklist is provided (see additional file 2). Data generated within the toolkit platform by the implementation teams whilst working with the toolkit is used to provide an overview of the projects, solely for the purpose of contextualising the qualitative data reported. Full details of trial data collection are published in the study protocol [15].

Ethical approval for the process evaluation was granted by the University of Northumbria, UK.

### Study participants and recruitment

The study settings were thirteen sites in the IMA project that used the toolkit, and the sites were aware they would be taking part in the qualitative process evaluation from the outset of the IMA project. Site participation in the qualitative data collection was initiated by the research team contacted the site Implementation Lead (IL), soon after the site had been informed of their entry point into the intervention phase of the trial (three months ahead). Twelve of the sites were included in the effectiveness study ([17] for more information about the trial sites). An additional site intended for inclusion in the trial, and trained to use the toolkit, did not meet trial study requirements at cross-over, and was included in the process evaluation only.

Study participants were members of the site implementation teams: Implementation Leads (ILs), core team members who were part of the teams and working closely with ILs, and other individuals involved in local implementation work. These ‘other’ individuals were stakeholders who had key roles in relation to the work undertaken through the ItFits-toolkit, for example, as staff in partner organisations to the sites, IT specialists, or communication experts. The research team worked with each site IL to identify and approach appropriate team members and stakeholders for interview.

### Data collection and theoretical approach

This qualitative study was informed by Normalisation Process Theory (NPT), a middle-range theory that explains the implementation, integration and embedding of new interventions and practices [18]. Focusing on how implementation is achieved through the *collaborative work of participants* in an implementation process, NPT is ideally suited to this investigation of how implementation teams undertake tailored implementation. Qualitative data was collected to understand how Implementation teams engaged with ItFits-toolkit, and the factors that shaped tailored implementation work via ItFits-toolkit in the context of the trial.

#### Interviews

Were conducted using an NPT-informed topic guide (see Additional file 3) and informed by prior interviews and by our developing analytic work as the study progressed. They were also informed by reviewing the data from the online platform (e.g., current position in the process, chosen barriers, etc.), prior to or during the interviews. Interviews were conducted mostly on an individual basis, but some included multiple team members.

Overall, 55 interviews were conducted with 30 individual participants across the study duration. Some interviews included more than one interviewee. Some interviewees took part in more than one interview. Interviewees were Implementation Leads (ILs) (*n* = 19), core team members (*n* = 9), and other stakeholders (*n* = 2). The number of ILs exceeds the number of sites due to staff turnover. Details of interview participation by site are provided in Table 3.

**Table 3:** Interview participation by site

Sites	Interviews [*n*]	Interviewees [*n*] *(number of interviews they participated in)*			
		ILs	Core Team Members	Stake-holders	Total Participants
S1	5	1 *(5)*	-	-	1
S2	6	1 *(4)*	-	2 *(2)*	3
S3	4	3 *(7)*	1 *(3)*	-	4
S4	2	2 *(2)*	-	-	2
S5	6	2 *(5)*	2 *(1)*	-	4
S6	4	1 *(3)*	1 *(1)*	-	2
S7	3	1 *(3)*	-	-	1
S8	3	1 *(3)*	-	-	1
S9	3	2 *(5)*	-	-	2
S10	7	2 *(4)*	4 *(3)*	-	6
S11	3	1 *(3)*	-	-	1
S12	6	1 *(6)*	1 *(1)*	-	2
S13	3	1 *(3)*	-	-	1
	**55**	**19**	**9**	**2**	**30**

Observational data was also collected, relating to meetings and events related to use of the ItFits-toolkit. Meetings for observation included introductory sessions, monthly group (multisite involving participants in toolkit active phase) support calls, and site-specific follow up calls. These meetings were undertaken either face-to-face or via teleconferencing and were facilitated by project members outside the process evaluation team. Monthly support calls with implementation teams provided an opportunity to monitor progress and flag any emerging technical issues users experienced when using the ItFits-toolkit. Follow up calls (1 and 3 months after the toolkit exposure period) were offered by the support team as implementation work initiated through ItFits-toolkit continued in some sites. Data was available to the process evaluation team from 19 of these calls involving implementation sites: monthly support calls (*n* = 9); one month follow-up support calls (*n* = 5), and three-month follow-up support calls (*n* = 5) (see Table 4). Support call facilitators took detailed minutes during these 19 calls, which were subsequently anonymised and coded by the process evaluation team. The total number of sites participating in each of the recorded calls ranged from 1–5. In some cases, the process evaluation team members observed these calls in real time and developed additional fieldnotes that were also analysed where available.

**Table 4:** Participation in support meetings (calls) by site

	**Number of calls that sites participated in**		
**Site**	**Monthly support calls**	**1 month follow-up calls**	**3-month follow-up calls**
S1	2	1	1
S2	2	-	1
S3	2	1	-
S4	3	-	-
S5	3	1	-
S6	1	-	-
S7	2	-	1
S8	1	-	-
S9	2	1	1
S10	2	-	1
S11	1	1	1
S12	2	1	-
S13	2	1	1
**Total number site participation**	**25**	**7**	**7**
**Total number documented calls**	**9**	**5**	**5**

#### Data management

All data were securely managed, with password protection. Interviews were audio-recorded with participants’ consent and transcribed verbatim for analysis. Fieldnotes were electronically recorded as typed documents. All data were carefully anonymised to prevent identification of either the individual participant or the participating study site. To preserve site anonymity, an electronically generated random number sequence was used for site identifiers for reporting in this paper, that do not reflect order of entry into the toolkit phase of the trial, and therefore also do not correspond to site numbers used in the trial results paper [17]. Qualitative software (NVivo) was used to support the data management and analysis process and establish an audit trail.

#### Qualitative data analysis

Our data analytic approach was inductive, informed by principles of first and third generation Grounded Theory [26]. It included standard procedures of rigorous qualitative analysis, including pre-coding, open and focused coding, constant comparison [27], memoing, tables, diagrams, and deviant case analysis [28] were used with the qualitative data. Initially, we worked primarily with interview data (via transcripts), cross-checking and comparing with data collected in minutes from support calls and our own fieldnotes to gain deeper understanding of activities in each site. A team-based approach to data interpretation, coding, and analysis ensured that a range of possible interpretations of data could be explored. Practically, this involved individually and collectively to read and pre-coding documents, then more formally starting a process of open (and over time focused) coding them, adapting the codes as new data emerged. It also involved thinking with the data and/or codes in a range of ways, for example, writing memos about key emerging issues, creating diagrams to show potential relations between codes. Working from earlier stages of coding, progressing through to the identification of ‘topics’ (sometimes described as categories or themes), key analytical ideas about tailored implementation and use of the ItFits-toolkit were developed through regular research team-based analysis workshops. These workshops, held approximately every 4–6 weeks during the main analysis period, involved researchers from other work packages in the ImpleMentAll project, who had different perspectives on the contexts of the sites and the activities they were undertaking from their roles in delivering toolkit training and guidance, collecting data from sites about the pre-intervention period, and working with sites for effectiveness trial data collection. These additional participants (outside the process evaluation team) worked with carefully anonymised data but their insights facilitated the analytic process.

In parallel with topic-based analysis across the full dataset, we undertook site-level analyses. In contrast to topic-based analyses, these site-level analyses allowed us to take a more longitudinal approach to developing understanding of each site’s progression through the toolkit over time (from multiple time point interviews), including the different roles of individuals in the process, the ways in which they worked with the toolkit, and the unique contextual factors that shaped their work. Site-level analyses supported ongoing development of the coding and organisation of data across the different topics, as insights gained from site-level analyses were fed back into our overall analytical process.

Analysis occurred concurrently with data collection following the stepped order of trial sites’ cross-over to the ItFits-toolkit condition. This allowed for emerging ideas and concepts found in earlier rounds of fieldwork to be explored in subsequent ones *(theoretical sampling)*. It also enabled moving from team-generated data coding, to developing higher order understandings of the processes being described through the data by exploring patterns and differences across and between study sites. The key topics developed through our analytic process are included in Additional file 4. In this paper, we draw from the full analysis to answer the more focused question of how implementation teams developed and undertook their tailored projects whilst using ItFits-toolkit.

## Results

The results of the qualitative analysis explored in detail how implementation teams worked through the toolkit to undertake tailored implementation in relation to (1) developing goals and the focus of their projects; (2) matching strategies to determinants; (3) executing the projects they designed, and (4) reflecting on the impact of their strategies. We also present data on (5): how the context of the effectiveness trial may have shaped the conduct of tailored implementation work in the ImpleMentAll study.

### Project goals and focus

Sites varied in how they approached setting goals and determining the focus of their projects. Within sites that developed multiple projects, some projects aimed to address different goals and barriers, or to address a common barrier across projects but with different strategies, or to undertake similar projects but with different stakeholder groups. This first step was not always straight forward. Agreeing on goals and focus for implementation projects ‘take time’:

…it took quite some time to (…) find agreement on this, because like the ICT guys, they want to improve the usability of the system, but yeah, but for us it’s we just want patients to use the system. So it was a bit of a discussion, so it took us, I think, two meetings. [Site 13]

Developing goals and a focus for projects to be developed using ItFits-toolkit took negotiation. The project-related goals generally aligned with the trial’s primary aim of increasing service uptake. The concept of ‘uptake’ however involves consideration of different points along a trajectory from initial awareness to completing therapy programmes. Sites’ implementation projects targeted different points in this process – for example, some were targeting initial referral and engagement; others were working on increasing the conversion rate from expression of interest to commencing therapy.

For some sites, facilitating staff engagement with the iCBT service was seen as an essential first step to achieving increased service uptake. For example, one site approached this by working on the usability of the iCBT platform and technical integration alongside assessing local needs from a clinician perspective, for developing more targeted strategies. In contrast, another site worked on increasing the proportion of ‘treatable’ patients coming through the registration process. Such an approach may not align with outcomes related to increasing service uptake yet could potentially impact positively on therapy completion.

Choices made about goals should be understood with reference to the ways in which ItFits-toolkit is designed – to get implementation teams to ‘focus on one thing at a time’, while also engaging in stakeholder discussions and designing projects collectively. In one site, the team were initially interested in (and planning to undertake) a ‘project on reach’. After an initial core team meeting, they decided to focus on patient suitability, as this would align better with the preferences of the therapists involved in the service. After that project ended, they set up a new project on reach. Here, there seemed to be a tension – and balance to be achieved—between staying within the focus of the trial (increasing uptake) and working on problems that are central to certain stakeholders. The toolkit’s requirement to choose one goal to focus on (*P2 ‘be focused’*), was also seen as a challenge for some. One team noted that:

We have to choose at the beginning three goals, and for each goal three barriers, and at the end we have to choose only one…and we wanted to keep all of them, so at the end finally what we are going to do with in any case we will work on the goals we had. It’s just that finally we have one large goal [Site 3]

This team chose the highest-level goal, and then developed a multi-faceted strategy to represent the different goals initially established in Module 1. The teams, at times, found ‘work around’ solutions to navigating the needs of the IMA trial and the ItFits-toolkit process.

### Matching barriers and strategies

Working to engage with a range of stakeholders to generate and verify ideas, was considered a key driver of the matching process. Most of the implementers described how the initial discussions and engagement with stakeholders resulted in a broad range of barriers. They could also be used to check whether they were addressing problems that were relevant to them:

And then we wanted to double check if we’re going into the right direction, so I created this very small survey and sent it out to the [therapists] to collect their opinions if this is really the important next step or if we are on the […] if we’re moving to the wrong direction. And actually, it fits really well […] Yeah, so our goals were not changed through the survey results [Site 7]

So, working with stakeholders in this way, supported implementers in the process of narrowing down their focus, translating their broad ideas into more specific solutions. Engaging with them increased confidence in the implementation activities that they were designing.

They also described how various steps and principles embedded in the toolkit helped them match one barrier to a specific strategy.

I think the toolkit has been very helpful in limiting us, like really narrowing it down. We had lots of ideas in the beginning […]. But when the […] barriers are chosen [it was then] that we really…we really saw that it’s important to be concrete and (..) yeah, I think it helps narrowing us down in our way towards the strategy so that we didn’t just pick, pick and choose everything that we could think of. [Site 2]

This narrowing process was perceived as useful, in part as it was different from implementers’ normal way of working. Implementers liked that the toolkit provided them with both the content – repositories of barriers and strategies – and the technical instructions for carrying out the matching work. In one site, the list of strategies in the toolkit helped them to ‘think outside the box’ *(P3 ‘be different’)* to consider ‘approaches that I think we would not have usually come up with’ [Site 7]. The pre-specified lists of barriers and strategies for selection were generally considered sufficient and ‘workable’. Some sites did however need to use the toolkit’s option to *customise* and add their own barriers or strategies to their problem of focus (listed in Table 2).

At another, a strategy to address incoming legal changes, needed faster action and progress than the toolkit would normally suggest – so the iterative cycles of stakeholder engagement were not undertaken.

### Planning and executing implementation projects

Sites acknowledged that developing strategies and executing projects takes time. At some sites, it was the engagement work with stakeholders on the ground that was labour- and time- intensive. At others, the project work, like adding new content to an existing iCBT platform, ‘took quite some time’. Unexpected challenges sometimes emerged. Despite early involvement of relevant stakeholders, one site discovered that the production and dissemination of new promotional materials they had developed required additional organisational approval.

[We] were not aware that we need an official permission. So, we thought we only have to show it to inform, to present it, but not that it is required as official permission. [Site 10]

Gaining that official permission took an extended period of time. At other sites, problems identified at a later stage through working with stakeholders could be more easily accommodated. For example, a solution that had early direction and backing from stakeholders turned out to not align with their needs.

So it was kind of crazy because you were in contact so much with each other “and yes, this is what we need” but then in the end it seemed like the process was just a little different and it was not exactly what they need […]. [Site 6]

The team ended up shifting their focus. The ItFits-toolkit process was designed to allow for, and to encourage, the adaptation of implementation projects (*P6 ‘be flexible’*) as they progressed. Some sites also achieved this either by running multiple projects in parallel within the toolkit, or by finishing projects and initiating new ones with a different focus.

The sites’ implementation projects were also influenced by a range of situational factors that were outside of their direct control. These included earthquakes, coronavirus pandemic, and extensive bushfires that caused major disruption to regular service provision in some of the sites, as well as routine ‘holiday periods’ (summer, festive holidays, school holidays) where delays in progressing project plans were anticipated or experienced. The coronavirus pandemic directly influenced many projects. Some impacts were potentially positive, as iCBT services at some sites gained more relevance to target participants under periods of (face-to-face) contact restriction. However, those who relied on referral processes taking place in service settings (such as general practices) were negatively impacted. The pandemic often included a re-prioritisation of the work of those involved in providing care and services during the pandemic. The implementation teams recognised that some of their intended plans and strategies might not be possible to progress during that period. The toolkit allowed teams to adapt and re-prioritise what they focused on, which is what some teams did.

Finally, changes within the implementation teams themselves and their organisations became a limiting factor in some sites. Here, issues relating to staff capacity, staff turnover and changes in management impacted on decisions about the number of projects that teams undertook or delayed or halted specific projects altogether. For example, a site reported dropping one of their (three) planned toolkit projects as a direct result of a team member going on long term leave; in another, an important ‘problem area’ identified by the implementation team was seen as unlikely to be progressed under an incoming manager at the site.

### Reflecting on impact

In Module 4, the implementation teams were prompted to make self-assessment of the impact of the strategies they had implemented. Some teams could see some immediate impacts of their work. At Site 3, it was observed that their work to develop ‘nicely made’ guides and resources for practitioners in the iCBT service were well received, and that they found the practitioners to be ‘motivated’ as a result. In another site, face to face training was thought to have increased the numbers of general practitioners referring patients to their service. However, sites generally expressed that within the six exposure months, there ‘hasn’t been enough time’ to observe any impacts from their toolkit projects in terms of ultimate goals of increased service uptake.

Expectations of impact on referral or uptake rates, was at times tempered by a sense of modesty as to the scope of the implementation projects that were possible. One respondent explained that ‘I’m not that optimistic […] I think you need a bigger [awareness raising] campaign’ (Site 10). Due to a range of external constraints, including partner organisations’ policies, the project did not have the scope intended – instead it was kept ‘modest’. Discussion in another site centred on two projects being developed with the toolkit, both focused on increasing referrals. One targeted psychologists, the other on general practitioners and nurses:

I am not sure yet to say how much [impact we] have, for example, in increasing number of referrals, but I think that in overall it was good for us because gives us an impression of what we might do better, what we might change in our activities, implementation as usual. [Site 12]

So, despite such positive assessment—that both these projects enabled a ‘learning process’, a focus on ‘what they might do better’—an increase in referrals and uptake could not be assumed.

For several sites, a lack of access to data to make confident evaluations limited their ability to assess impact as instructed in the toolkit. Some found that gaining access to service level data for assessing progress in relation to metrics like conversion rates (from screening/referral), service uptake, and programme completion was more difficult. For example, one site directly received the numbers of patients registered to use the iCBT platform directly and had basic information about those patients. However, they could not access the numbers of patients engaged in therapy or information about how many sessions they completed, as ‘it was very difficult to get this kind of information by our therapists’ (Site 9). As such, they could not ‘confirm’ the anecdotal information they were gaining. Generally, sites used a more multi-faceted approach to evaluating the impact of their implementation work. Alongside accessing service data, many sites undertook surveys (a functionality of the toolkit), discussions with informants, or even more formal ‘qualitative’ research.

### Tailored implementation work in the context of the IMA trial

The implementation work undertaken with ItFits-toolkit was also shaped by the very reason for initially engaging with this process, that this work was taking place within a trial. For example, some mechanisms were built into the toolkit, in part, to enable better data collection from the toolkit within the trial. This included ‘movement’ restrictions—being unable to move back and forth through modules and change ideas and responses – and progression restrictions—separating out of the work into ‘one step at a time’. This was not ideal for some:

[F]or example, we have two goals, which I think maybe they need to be modified or we can edit a little bit, not change them but just edit them, and I don’t think I can go back, for example, to goals and then review the barriers and then go further. [Site 9]

People at several sites expressed a desire for ‘reading ahead’ through the toolkit, to anticipate upcoming work and to allow more efficient planning of work with the implementation team. Although this was possible to an extent and advised during training, some either didn’t see this or found it limited. For some, the separation of Modules 1 (about barriers) and 2 (about strategies) and the inability to move back forth between them and adjust them was counter intuitive. However, several teams undertook multiple projects in the toolkit in parallel, so there was scope to create ‘work arounds’ where teams wanted to work on different strategies at the same time. Notably, the six-month period that sites were asked to complete the full toolkit programme through to the end of Module 4, was reported as challenging. Such a timeline was seen, by some, as ‘too short’ to collaboratively develop and introduce new strategies, let alone see and evaluate any impact from the strategies they implemented.

Although for some, the trial timeline and allocation of resources for implementation work was seen as ‘motivating’, the pace of working through the toolkit modules was slowed down. Some teams reflected that the trial provided research-driven constraints around the way they worked:

If it was an effectiveness trial, we wouldn’t change things [about the service]. [...] if we were trying to implement the service then […] it would be a different process again, I think. We would be trying to (.) constantly and rapidly optimise how we do the implementation. (.) But I think we want to do it systematically and carefully in the context of the trial because we want to know, we want to document what we’re doing to see what effect that is having rather than just trying to throw everything at it and not research them as systematically. [Site 5]

In this way, the broader trial context, alongside the ideas embedded in the toolkit (*P1 ‘be pragmatic’; P2 ‘be focused’*) changed what they were doing with regards to the iCBT service implementation. However, this raises questions around sustainability of this slower, more methodical approach. As one Implementation Lead noted,

So, if you are part of a trial and you know “OK now you are under this intervention phase”, you want to use [ItFits-toolkit], and we are using it. And we were very engaged in this and motivated and so on, but it was not like “oh now we have in our company a new ItFits-toolkit, it’s part of our processes” […] so it was not the perception that it is a normal workflow in our company. [Site 10]

This was clearly ‘different’ to everyday implementation work. Out with the motivating context of the trial, at this site, ItFits-toolkit was yet to be seen as routine, every day and normal implementation work.

## Discussion

In this paper, we sought to explore how implementation teams undertake tailored implementation using a self-guided toolkit in the context of the ImpleMentAll study, where implementation was targeted at improving the uptake of iCBT and was undertaken by teams working in service organisations. Our findings are discussed in relation to the empirical and conceptual literature on tailoring, and with reference to the findings of the ImpleMentAll effectiveness study [17].

Aligning with current conceptualisations of implementation tailoring [9, 13], our results show that ItFits-toolkit supports an implementation process that includes barriers assessment, matching of implementation strategies to identified barriers, developing and detailing implementation strategy plans, and strategy execution, and strategy assessment. We had designed ItFits-toolkit to address Wensing’s [11] recommendations for implementation tailoring to include (1) greater stakeholder engagement in the tailoring processes than is typically involved *(P4 ‘be open’)*, and (2) to make greater use of continuous monitoring and adaptation of approaches (*P6 ‘be flexible’*). Despite the structured approach that ItFits-toolkit provided, considerable flexibility in how teams could use ItFits-toolkit was designed into most of the process, and evident in their engagement with it. Tailoring – and responsiveness in relation to local contexts and processes – was evident in variability of the number and succession of projects the teams initiated, the specific goals of their projects, and in their innovative approaches and adaptability to challenges as encountered. In short: the teams’ engagement with ItFits-toolkit demonstrated ‘fidelity of enactment’ [29] in relation to the guiding principles embedded within it.

Further advancing the agenda set out by Powell et al. and others [12, 30], ItFits-toolkit also offers a way of improving the tracking and reporting of implementation strategies. In the trial, self-reported ‘effort’ (logged time input) did not increase significantly when teams moved from pre-toolkit (implementation as usual) phase to the toolkit phase of the trial [17], suggesting that the toolkit itself is not burdensome. The data captured in the teams’ toolkit projects, not only gave us (as researchers) insights about the teams’ approaches to tailoring that were explored directly in qualitative fieldwork for the evaluation, but it also enabled teams to reflect on their activities and decisions, thus facilitating ongoing implementation work. Our study therefore makes important steps towards addressing McHugh et al.’s questions about what tailoring constitutes, how it might work, who and what it involves, and how it should be evaluated [13].

Our examination of tailored implementation in (real) service settings is a further advancement of knowledge. In relation to stakeholder engagement, our evaluation demonstrated how teams undertook and experienced iterative cycles of this to support implementation work, across different phases of the tailoring process. Although asking teams to engage with stakeholders in each module, ItFits-toolkit did not dictate how it should be done, or who should be engaged with: these were decisions made by the teams, working to improve the uptake of real services. In another paper from this study, we offer an initial model of stakeholder engagement for implementation, I-STEM, that can be used as a guide for planning or evaluating stakeholder engagement in implementation projects [31]. In relation to implementation strategies themselves, differences in how these are conceptualised and operationalised ‘on the ground’ are likely. In our study, some teams created custom strategies that were not seen to be represented in the list derived from ERIC, which has also been found in other studies of use of implementation strategies in real world settings where ‘new’ strategies not included in ERIC have been proposed [32, 33]. In real world implementation, further understanding of how strategies employed change over time from initial planning, to executing, and maintaining service innovations is needed [30].

Our study also advances theoretical understanding of mechanisms of Normalisation Process Theory (NPT) [18, 34] and how these relate to tailored implementation. NPT informed both the development of ItFits-toolkit, and our approach to data collection and analysis in this qualitative study: as such, we report findings of collective implementation work ‘in action’. We suggest our study further advances understanding of two NPT mechanisms that are *central to tailored implementation*: (1) workability, and (2) reflexive monitoring. NPT proposes that successful implementation requires ‘integrational workability’, in that participants in the implementation process can make it ‘work’ by adapting and coming up with creative solutions to problems as they arise. Our data revealed how the ItFits-toolkit provided implementers with a structure for their work, but with space to create workability between the toolkit, themselves as a team, and demands of their work so that they could use it to advance their priorities for service implementation. In tailored implementation, this quality of workability is inextricably linked with reflexive monitoring, which refers to the ability to appraise and reflect on implementation activities and their effects, and to make modifications and support sustained engagement with the implementation process. Our findings demonstrate how implementation teams engaged in cycles of reflexive monitoring – through stages of barrier identification (e.g. prioritising with stakeholders), matching of strategies to barriers (e.g. making assessments of feasibility and effectiveness), designing implementation plans (including defining criteria for success), and evaluating the impact of their strategies. Reflexive monitoring is thus a key embedded mechanism across the ItFits-toolkit process, and tailored implementation more generally.

Although not the primary focus of this paper, our study also allows some reflection on the primary finding of the ImpleMentAll trial: that there was a small positive effect of ItFits-toolkit on the primary outcome of iCBT ‘normalisation’ [15]. Firstly, the scope of most projects developed using ItFits-toolkit was relatively modest. Many focused on educational strategies that have low median effects on improvement in care [12], and targeted discrete elements of – or steps within—overall plans the teams had to improve referrals or uptake of their iCBT services. The trial found no effects for the toolkit on secondary outcomes of uptake and referrals to the iCBT services, which might be considered as more ‘distal’ implementation outcomes [35]. However, effects of educational interventions may be more ‘proximal outcomes’, readily detected in the measure of normalisation (NoMAD [20]), where staff participants in the implementation process being surveyed have likely been exposed to such interventions. Secondly, our participants described how tailored implementation ‘takes time’. Here, time is in terms of planning and executing tailored implementation projects, as well as in terms of any impacts becoming evident. Whilst this is both intuitive, and evidenced [36] our study serves as an important reminder of this, which has implications for evaluating implementation interventions. Insufficient timeframes for outcomes was one possible explanation offered for lack of effectiveness of tailored implementation in the TICD study [11]. Thirdly, despite teams demonstrating considerable adaptability, external influences that were outside the control of the teams affected their capacity to progress and complete their projects as intended, and in some instances, obstacles could not be overcome, and planned projects could not be completed. Where services were themselves disrupted – whether through the coronavirus pandemic, natural disasters, or other causes – so too were their implementation projects. Finally, although the implementation work was conducted in real service settings, implementers were aware of how their implementation decisions and activities were shaped by working within a research trial. The trial itself thus shaped in some ways, how tailored implementation work was approached. This paper aimed to demonstrate *how* implementation teams undertook tailored implementation using the ItFits-toolkit, rather than to explain the outcome of the effectiveness trial. Our findings contribute initial insights towards the latter, but full explanation of the trial outcome requires further analytical investigation at the project level.

### Strengths and limitations

A limitation is that our qualitative work focused more on engagement with the toolkit across study sites, than on exploring in greater detail, the micro-level detail around the decisions the teams made during the toolkit process. However, given the scale of this study, our approach of using the toolkit data in real time to inform interviews has provided the best possible solution to understanding the tailoring undertaken by the teams in ImpleMentAll. Our focus on facilitating implementation in real service settings, means that the positioning of this work within an implementation study was both a strength and a limitation. The supportive structure that the trial gave to implementation teams was key to their engagement with ItFits-toolkit over an extended period, allowing us to observe in detail ‘the work’ of tailored implementation. However, although participants were generally positive about their experiences of working with the toolkit, engagement with the toolkit under ‘natural’ (non-research) conditions would differ and merits further exploration. The level of diversity of context and participants (services, practitioners, and implementers) that the ImpleMentAll study provided, served as a valuable testbed for ‘proof of concept’ for a self-guided approach to tailored implementation in service settings. At this level, our study findings are relevant for others who are developing and evaluating similar approaches to tailored implementation in service settings. We therefore offer a set of suggestions that might further progress both research and practice in this field (Table 5). The toolkit itself, although representing a transferable approach to tailored implementation and being freely available for use (https://itfits-toolkit.com/), is currently limited by its content being written (using examples) for the implementation of electronic mental health services. This will be addressed in future development work, but for further information on the ItFits-toolkit see Additional file 5.

**Table 5:** Suggestions for tailored implementation in service settings

Suggestions
1. Facilitate a team based collaborative approach to implementation
2. Ensure dedicated time for team members to engage in implementation activity alongside or as part of service delivery or other work
3. Provide materials to guide and support tailoring (goals, barriers, strategies, plans) that serve as prompts, yet can be used flexibly
4. Involve core stakeholders in the design, delivery, and evaluation of strategies
5. Provide guidance in identifying and engaging with stakeholders across stages of implementation
6. Take a structured approach, allowing time for collective reflection and analysis of progress and at regular time points
7. Support prioritisation of ‘one step at a time’ to build progress over time using pre-defined success criteria
8. Allow sufficient flexibility to accommodate parallel or sequential projects
9. Allow sufficient (process) flexibility to accommodate emerging contextual factors affecting implementation
10. Allow sufficient time for strategies to be designed, delivered and evaluated
11. Involve continuous monitoring and adaptation of strategies (iterative cycles)
12. Ensure timely access to service level and other data needed for impact assessment and adaptation of implementation strategies

## Conclusion

Implementation science-informed, self-guided tailored implementation in service settings enables implementers to undertake tailoring within their organisations. The ItFits-toolkit is intended for use by implementers working in service implementation roles, ideally in teams, and provides a structured process with resources and guidance to help focus implementation activity towards achieving collectively agreed implementation goals. In our study, it enabled implementation teams to work on these goals, by determining and prioritising barriers needing to be addressed, and by selecting, developing, and executing implementation strategies matched to the barriers they had identified. Our study showed that, in this context, implementation tailoring involves detailed work, is supported through iterative cycles of stakeholder engagement, and takes time.

### Supplementary Information

Supplementary Material 1. Standards for Reporting Implementation Studies (StaRI) checklist.

Supplementary Material 2. Standards for Reporting Qualitative Research (SRQR) checklist.

Supplementary Material 3. Interview topic guide.

Supplementary Material 4. ImpleMentAll qualitative process evaluation topic summary.

Supplementary Material 5. Snapshot – The ItFits-toolkit.

## Data Availability

The datasets generated and/or analysed during the current study are not publicly available because this would likely compromise participants’ anonymity, particularly to others involved in the ImpleMentAll project. Some descriptive data may be available from the corresponding author on reasonable request.

## Abbreviations

iCBT: Internet-based Cognitive Behavioural Therapy
ERIC: Expert Recommendations for Implementing Change
IMA: ImpleMentAll project
APPEASE: Acceptability, Practicability, Effectiveness, Affordability, Side-effects, and Equity (framework)
TIDieR: Template for Implementation Description and Reporting
ILs: Implementation Leads
I-STEM: Implementation STakeholder Engagement Model
NoMAD: Normalisation Measure Development
TICD: Tailored Implementation for Chronic Disease (Study)
