# Supplementary material for: How is tailored implementation undertaken using a self-guided toolkit? Qualitative study of the ItFits-toolkit in the ImpleMentAll project
Source: Implement Sci. 2024 Jul 11;19:48. doi: 10.1186/s13012-024-01380-w (PMC11241992; doi:10.1186/s13012-024-01380-w)
Supplement: Supplementary file 3 — Supplementary Material 3. Interview topic guide. [file 13012_2024_1380_MOESM3_ESM.docx]

# Additional file 3: Example Interview Topic Guide

*Note: The interview schedule is developmental and will change over time. The questions will need to be tailored to the specific answers of each interviewee. The questions will also be developed from the findings of prior interviews, as well as observations. The interview schedule given here is therefore a general topic guide for the qualitative interviews.*

**Introduction**

- Explain purpose of the study and this interview
- Explain interview recorded but details will be confidential
- Explore any questions or concerns?
- Re-affirm consent

**About your role**

Could you tell me about your role in the organization?

- Job title
- Responsibilities

**Clinical service**

Could you tell me a bit about the iCBT service you trying to implement? [*Compare to data we already have*]

- Evidence-based
- People involved
- Stage of implementation

**Implementation as usual**

Do you remember what you did to improve your service before receiving the ItFits-toolkit toolkit? [*Compare with data we already have*]

- Setting up the service for the trial
- Improving implementation of service
- Timing of effort
- Stakeholder engagement
- Barriers
- Successes

**Minimal guidance**

Could you tell me a bit about the guidance you received to support your use of the ItFits-toolkit toolkit?

- Introductory session
- Monthly group support calls
- Closing session
- Ad-hoc technical assistance

**Implementation with ItFits-toolkit**

Can you walk me through how you have been using the ItFits-toolkit toolkit so far?

- People involved (roles/ understanding/ commitment/ enrolment)
- Likes/ dislikes
- Changes in working approach
- Principles
- Implementation project(s)
- Goals and barriers (identification/ prioritization)
- Stakeholder engagement

**Follow-up interviews at T2 and T3** [*Note that this will depend on the progress that sites have made*]

- Strategies
- TIDieR
- Sub projects
- Plan to assess impact
- Monitor
- Review (reflections)

**Individual user experience of ItFits-toolkit**

Since you started using ItFits-toolkit, how many times have you used it?

- Motivations
- Expected outcomes
- Planning
- Barriers/ facilitators
- Coping planning
- Routines/ habit
