# Supplementary material for: How is tailored implementation undertaken using a self-guided toolkit? Qualitative study of the ItFits-toolkit in the ImpleMentAll project
Source: Implement Sci. 2024 Jul 11;19:48. doi: 10.1186/s13012-024-01380-w (PMC11241992; doi:10.1186/s13012-024-01380-w)
Supplement: Supplementary file 4 — Supplementary Material 4. ImpleMentAll qualitative process evaluation topic summary. [file 13012_2024_1380_MOESM4_ESM.docx]

**Additional file 4: ImpleMentAll Qualitative Process Evaluation topic summary**

| **Topic number** | **Topics** | **Contribution** |
| --- | --- | --- |
| T1 | Working with the toolkit – introducing & embedding | Understand the different approaches, ***work flows and processes; co-ordination work; appraisals*** of the process |
| T2 | Working with the toolkit – negotiating compromises | Understand how toolkit work is ***undertaken collectively*** (in teams/ organizational context) and ***in response to constraints*** |
| T3 | Matching (barriers to strategies) *(modules 1 & 2)* | Understand how the toolkit **facilitates and constrains choices of strategies in relation to barriers** |
| T4 | Rolling out and monitoring *(modules 3 & 4)* | Understand how the toolkit facilitates and constrains the ***implementation and assessment of strategies***; and ***effects of external factors*** on implementation projects |
| T5 | Trial related factors – outcomes and temporal issues | Understanding of how the IMA trial context shapes ItFits work: alignment of projects with trial objectives; trial timelines and the ‘real world’ |
| T6 | Going forward – taking the learning forward | Identifying transferable ‘learning’ from the ItFits process and how it might be applied in future implementation work |
| T7 | Improvements (to ItFits toolkit) | Suggestions for improvement to process and functionality and/or different versions for different uses and purposes |
