# Supplementary material for: How is tailored implementation undertaken using a self-guided toolkit? Qualitative study of the ItFits-toolkit in the ImpleMentAll project
Source: Implement Sci. 2024 Jul 11;19:48. doi: 10.1186/s13012-024-01380-w (PMC11241992; doi:10.1186/s13012-024-01380-w)
Supplement: Supplementary file 5 — Supplementary Material 5. Snapshot – The ItFits-toolkit. [file 13012_2024_1380_MOESM5_ESM.pdf]

GETTING EHEALTH IMPLEMENTATION RIGHT

SNAPSHOT: THE ITFITS-TOOLKIT

ImpleMentAll

INTRODUCTION – WHO IS THIS SNAPSHOT FOR?

- Implementers facing barriers that mean slower or more costly than desired implementation of services and technology
- Health implementers delivering Internet-based Cognitive Behavioural Therapy (iCBT) – internet-based health care programs
- Implementers looking for an instrument to support tailored implementation in their next project
- Policy makers, decision makers, and organisations seeking to speed up the delivery of e-services and technology

WHAT WILL THIS SNAPSHOT TELL ME?

- How the ItFits-toolkit, a tailored implementation instrument, can support more effective and efficient implementation
- What iCBT implementers across eight countries, including low- and middle-income countries, learnt when using the ItFits-toolkit
- Headline recommendations from ImpleMentAll, a project systematically testing tailored implementation of eMental health services

The ImpleMentAll project received funding from the European Union's Horizon 2020 research and innovation programme, under the Grant Agreement No. 733025
The information in this document is provided as is and no guarantee or warranty is given that the information is fit for any particular purpose. The user thereof uses the information at its sole risk and liability. This content reflects only the author's view and the European Commission is not responsible for any use that may be made of the information it contains.

WHY IS IT IMPORTANT TO IMPROVE iCBT IMPLEMENTATION?

Recent research suggests that iCBT is effective for the prevention[i] and treatment[ii] of common mental disorders. Systematic reviews show comparable effects to face-to-face treatments in adults[iii]. iCBT for the treatment of depression and anxiety have shown to be effective when implemented in routine mental health care[iv]. Additionally, a recent meta-analysis showed that iCBT interventions for anxiety disorders can have significant effects in trials implemented in clinical care[v].

DID YOU KNOW that it takes about 15 years for effective eHealth tools to make it into routine care?

IMPLEMENTATION – WHAT ARE THE COMMON CHALLENGES?

eHealth interventions are not a new concept and large investments of time and funds have been expended on their development, testing, and implementation.

BUT - the road from an idea to routine care is still long and challenging for most interventions, and many do not make it at all.

Introducing innovations, such as iCBT, into routine health care systems is a complex endeavour and a lack of knowledge and implementation efforts around iCBT innovations has been identified as a challenge in the future direction of internet interventions[vi].

The ImpleMentAll project received funding from the European Union's Horizon 2020 research and innovation programme, under the Grant Agreement No. 733025
The information in this document is provided as is and no guarantee or warranty is given that the information is fit for any particular purpose. The user thereof uses the information at its sole risk and liability. This content reflects only the author's view and the European Commission is not responsible for any use that may be made of the information it contains.

IDENTIFYING BARRIERS AND ENABLERS TO IMPLEMENTATION

A systematic review[vii] that commenced in the MasterMind project and was published during IMA, sought to identify the determinants of practice (barriers and enablers) relevant to implementing eMental health for mood disorders in routine practice – this showed that the determinants could be clustered into six themes:

- acceptance • appropriateness • engagement • resources • work processes • leadership

WHICH BARRIERS WERE MOST PROBLEMATIC FOR IMPLEMENTERS?

In using the ItFits-toolkit, implementers are asked to select the three barriers most closely related with their implementation problem.

The top three barriers chosen by IMA implementation sites were:

- **Acceptance** – a lack of satisfaction amongst patients and staff with the iCBT services. Within this, discrete barriers to achieving 'acceptance' included a lack of education, lack of the necessary skills or competences, and a lack of awareness.

- **Working Processes** – a lack of direction and control of essential working process in relation to iCBT.

- **Engagement** – a lack of concrete structures and treatment plans.

The ImpleMentAll project received funding from the European Union's Horizon 2020 research and innovation programme, under the Grant Agreement No. 733025
The information in this document is provided as is and no guarantee or warranty is given that the information is fit for any particular purpose. The user thereof uses the information at its sole risk and liability. This content reflects only the author's view and the European Commission is not responsible for any use that may be made of the information it contains.

ImpleMentAll

WHAT IS TAILORED IMPLEMENTATION?

Tailored implementation is the identification of determinants of practice in a particular context or setting – this can include barriers, obstacles, problems, facilitators, enablers, and success factors. In tailored implementation, the identification of these determinants is followed by a selection of implementation strategies that directly address those determinants of practice.

Tailored implementation is fit for purpose, rather than a one size fits all approach.

WHAT IS THE ITFITS-TOOLKIT?

The Integrated Theory-based Framework for Intervention Tailoring Strategies!

The ItFits-toolkit (the toolkit) enables implementers to tailor implementation strategies and increase the normalisation and uptake of iCBT.

Through a digital platform, the toolkit provides implementers with guidance, resources, and support to identify and address key barriers to iCBT implementation.

It supports a team-based approach to developing and undertaking tailored implementation strategies, and consists of four key modules:

1 Identifying 2 Designing 3 Matching 4 Applying

The ItFits-toolkit is designed to work in a range of complex and diverse settings, organisations, and healthcare systems; and was developed to be structured but also flexible.

The ImpleMentAll project received funding from the European Union’s Horizon 2020 research and innovation programme, under the Grant Agreement No. 733025
The information in this document is provided as is and no guarantee or warranty is given that the information is fit for any particular purpose. The user thereof uses the information at its sole risk and liability. This content reflects only the author’s view and the European Commission is not responsible for any use that may be made of the information it contains.

FOUR MODULES OF THE ITFITS-TOOLKIT

1. **Identifying** and prioritising implementation goals and barriers to reaching these goals
2. **Matching** of implementation barriers to strategies
3. **Designing** a plan for carrying out strategies in a local context
4. **Applying** strategies and reviewing progress.

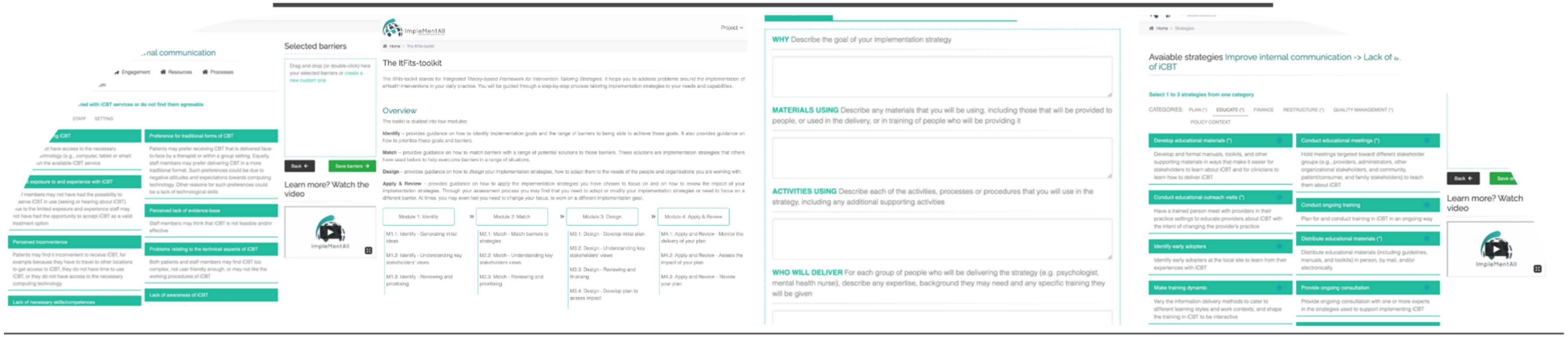

The ImpleMentAll project received funding from the European Union's Horizon 2020 research and innovation programme, under the Grant Agreement No. 733025
The information in this document is provided as is and no guarantee or warranty is given that the information is fit for any particular purpose. The user thereof uses the information at its sole risk and liability. This content reflects only the author's view and the European Commission is not responsible for any use that may be made of the information it contains.

THEORY, MECHANISMS, PRINCIPLES

The toolkit is based on concepts of implementation tailoring and is theoretically informed by Normalization Process Theory and Evidence-based Quality Improvement.

Work with and around the toolkit is based on **five core mechanisms:**

1. **Non-standardised, systematically guided step-by-step process**
2. **Stakeholder-based co-creation**
3. **Tools to identify local barriers, consult stakeholders, and match to suitable strategies**
4. **Evidence-informed materials on barriers, strategies, and intervention planning**
5. **Six working principles**

Use of the toolkit is guided by six principles:

Be pragmatic: Focus on realistic, achievable, next steps

Be focused: Focus on one thing at a time, don't try to do everything at once

Be different: Do not only focus on things that you feel most comfortable with or the things you would normally do

Be open: Listen to and value your stakeholders' knowledge and experience

Be organised: Each step needs an identified owner to take responsibility for delivery

Be flexible: The same solution may not work for everyone, so be prepared to adapt your plans and ideas

The ImpleMentAll project received funding from the European Union's Horizon 2020 research and innovation programme, under the Grant Agreement No. 733025
The information in this document is provided as is and no guarantee or warranty is given that the information is fit for any particular purpose. The user thereof uses the information at its sole risk and liability. This content reflects only the author's view and the European Commission is not responsible for any use that may be made of the information it contains.

ImpleMentAll

AS AN IMPLEMENTER, WHAT IS IT LIKE TO USE THE TOOLKIT?

The ItFits-toolkit was tested and evaluated across 13 implementation sites across eight countries – this is what they found:

- Implementers saw value in the toolkit to support and enhance their implementation work - it supported and complemented their existing implementation experience and knowledge
- It gave teams a structured and supported means of translating their implementation-related strategic intentions into action
- It helped generate and sustain a collective sense of purpose within their teams, it enabled more reflective discussions, challenged prior ways of work, and offered more structured and iterative approaches to implementation – they were able to reconfigure and adapt how they worked, using the toolkit with their existing organisational norms, logics, and routines
- The logic of the toolkit, its systematic and disciplined way of thinking and acting, breached and disrupted prior norms and routines and so enabled implementers to reflect on past implementation practices
- It institutionalised reflexive monitoring: putting ongoing testing, reflecting, and adapting courses of reasoning and action at the centre of the implementation process
- It facilitated engagement with stakeholders
- It introduced and enabled a (new) more co-ordinated relationship to aspects of implementation work.

"Because everything is in one place, we can view them in a systematic way, plus the details of what to do and how to do it. We have knowledge from before our projects, but here we have it systematically detailed and in one place. I think it's comprehensive and very, very helpful." (User from an implementation site)

The ImpleMentAll project received funding from the European Union's Horizon 2020 research and innovation programme, under the Grant Agreement No. 733025
The information in this document is provided as is and no guarantee or warranty is given that the information is fit for any particular purpose. The user thereof uses the information at its sole risk and liability. This content reflects only the author's view and the European Commission is not responsible for any use that may be made of the information it contains.

ImpleMentAll

MEET DENISE HANSSEN, AN ITFITS-TOOLKIT USER

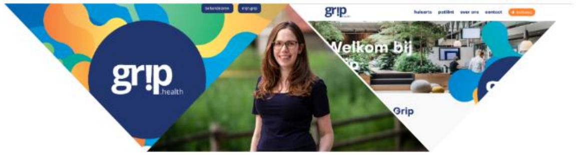

Where do you work and what eHealth project are you working on?

I work as a researcher and clinician (psychologist) at the University Medical Centre Groningen, in the north of the Netherlands. In recent years I have been involved in the development of 'Master Your Symptoms', a personalised, CBT-based eHealth treatment for patients with mild to moderate somatic symptom disorder. Together with an enthusiastic project group, we try to implement the intervention in general practices, and carry out scientific research to evaluate its use.

What are you trying to achieve through this project?

During the development of 'Master Your Symptoms', we continuously involved stakeholders such as general practitioners and patients. Nevertheless, we still found the implementation in clinical practice quite challenging. GPs are enthusiastic about the intervention, but in practice they hardly ever use it. We found this strange, as patients with somatic symptom disorder report low quality of life and GPs often find it difficult to provide an appropriate treatment. This was the main motivation for participating in ImpleMentAll: Could the ItFits-toolkit help us to improve the implementation process?

What makes implementation of this project challenging?

Before we used the ItFits-toolkit, we came up with all kinds of ways to promote implementation. We tried to creatively make GPs aware of Master Your Symptoms by making leaflets. We also created a beautiful website and a clear user manual. However, this had a very limited effect. From the moment we started using the ItFits-toolkit, the implementation process became more systematic and structured.

What differences did the ItFits-toolkit bring to your project?

So, the main difference is that we now have a clear implementation plan for 'Master Your Symptoms'! The toolkit has challenged us to identify with stakeholders the barriers we may face in implementing interventions for somatic symptom disorder. We even wrote an international scientific publication about it! We then used the toolkit to select appropriate implementation strategies to match these barriers, as well as to define a systematic evaluation of progress. Using the toolkit had the added advantage that, as a project group, we had to agree on which priorities we wanted to focus on. By doing so, I believe that we have become more unified as a team. There are still many challenges in implementing Master Your Symptoms in GP practices, especially with the COVID-19 pandemic. However, the toolkit has definitely given us direction on how to facilitate the implementation process in the future.

The ImpleMentAll project received funding from the European Union's Horizon 2020 research and innovation programme, under the Grant Agreement No. 733025
The information in this document is provided as is and no guarantee or warranty is given that the information is fit for any particular purpose. The user thereof uses the information at its sole risk and liability. This content reflects only the author's view and the European Commission is not responsible for any use that may be made of the information it contains.

WHO WILL BENEFIT FROM THE ITFITS-TOOLKIT?

The IMA project asked those within the consortium with first-hand experience and knowledge of the ItFits-toolkit, where future benefit of the toolkit lay, at the user but also at the organisational level.

Users

In terms of users, those in managerial or coordinating roles were deemed the most relevant user groups, such as implementation practitioners, project managers, team leaders, and hospital managers, as opposed to the frontline staff. This is due to their often more direct involvement in implementing new interventions.

Adding to these, researchers were also highly ranked in terms of relevance, which might link back to the current focus on knowledge valorisation and the fact that the toolkit is still in its early stages, requiring more research according to numerous consortium members.

Organisations

At the organisational level, those ranked the most relevant were hospitals and research institutions followed by Non-Government Organisations, consultancies, and regional authorities.

KEY RECOMMENDATIONS

1. **Improved implementation demands implementation strategies that incorporate both top-down and bottom-up approaches.**

2. **To deliver enhanced eHealth services and technologies to patients in need faster, policy makers and governments must invest more in strategies to drive and support innovation in the field.**

3. **For implementers on the front-line, a team approach is essential - an instrument such as the ItFits-toolkit facilitates implementation, but tailored implementation requires adequate and dedicated team working, commitment, resourcing, and allocated time.**

4. **The ItFits-toolkit pushes implementers to think and act differently. The six core working principles guide thinking toward a smooth workflow as well as precise and innovative results: *be pragmatic, be flexible, be focused, be open, be organised, be different.***

5. **In designing and delivering eHealth interventions, the perspective of the patient must always be maintained.**

The ImpleMentAll project received funding from the European Union's Horizon 2020 research and innovation programme, under the Grant Agreement No. 733025
The information in this document is provided as is and no guarantee or warranty is given that the information is fit for any particular purpose. The user thereof uses the information at its sole risk and liability. This content reflects only the author's view and the European Commission is not responsible for any use that may be made of the information it contains.

ABOUT ImpleMentAll

ImpleMentAll is a projected funded by the European Union that ran from 2017 until 2021. It aimed to solve one overarching problem: tackling the slow and inefficient uptake of eHealth interventions.

ImpleMentAll is a European collaboration towards faster and more effective implementation of eHealth interventions.

The project's raison d'être is founded on the notion that implementation of new services and technologies is time-consuming and costly – and often fails completely – not least in the healthcare domain.

The project has provided a gross wealth of information about tailored implementation of eMental health in routine healthcare in various contexts across the globe, including in a low- and middle-income country context.

No other project has thoroughly tested tailored implementation of eMental health services at this scale and systematically across different countries and health care contexts.

ImpleMentAll has been recognised as a successful project, and this success is celebrated by the partners who will keep applying ItFits beyond the original project's lifetime.

The ImpleMentAll project received funding from the European Union's Horizon 2020 research and innovation programme, under the Grant Agreement No. 733025
The information in this document is provided as is and no guarantee or warranty is given that the information is fit for any particular purpose. The user thereof uses the information at its sole risk and liability. This content reflects only the author's view and the European Commission is not responsible for any use that may be made of the information it contains.

FOR FURTHER INFORMATION

Please visit the ImpleMentAll Website – www.implementall.eu for a full list of partners, publications, resources, and further contact details.

Twitter: @EU_ImpleMentAll

The ImpleMentAll project received funding from the European Union's Horizon 2020 research and innovation programme, under the Grant Agreement No. 733025
The information in this document is provided as is and no guarantee or warranty is given that the information is fit for any particular purpose. The user thereof uses the information at its sole risk and liability. This content reflects only the author's view and the European Commission is not responsible for any use that may be made of the information it contains.

ImpleMentAll

REFERENCES

[i] Ebert, D. D., Cuijpers, P., Muñoz, R. F., & Baumeister, H. (2017). Prevention of Mental Health Disorders Using Internet- and Mobile-Based Interventions: A Narrative Review and Recommendations for Future Research. *Frontiers in Psychiatry*, *8*, 116. https://doi.org/https://doi.org/10.3389/fpsyt.2017.00116

[ii] Andersson, G., Titov, N., Dear, B. F., Rozental, A., & Carlbring, P. (2019). Internet-delivered psychological treatments: from innovation to implementation. *World Psychiatry*, *18*(1), 20–28. https://doi.org/10.1002/wps.20610

Andrews, G., Basu, A., Cuijpers, P., Craske, M. G., McEvoy, P., English, C. L., & Newby, J. M. (2018). Computer therapy for the anxiety and depression disorders is effective, acceptable and practical health care: An updated meta-analysis. *Journal of Anxiety Disorders*. Elsevier Ltd. https://doi.org/10.1016/j.janxdis.2018.01.001

Königbauer, J., Letsch, J., Doebler, P., Ebert, D. D., & Baumeister, H. (2017). Internet- and mobile-based depression interventions for people with diagnosed depression: A systematic review and meta-analysis. *Journal of Affective Disorders*, *223*(April), 28–40. https://doi.org/10.1016/j.jad.2017.07.021

Romijn, G., Batelaan, N., Kok, R., Koning, J., van Balkom, A., Titov, N., & Riper, H. (2019). Internet-Delivered Cognitive Behavioral Therapy for Anxiety Disorders in Open Community Versus Clinical Service Recruitment: Meta-Analysis. *Journal of Medical Internet Research*, *21*(4). https://doi.org/10.2196/11706

[iii] Carlbring, P., Andersson, G., Cuijpers, P., Riper, H., & Hedman-Lagerlöf, E. (2018). Internet-based vs. face-to-face cognitive behavior therapy for psychiatric and somatic disorders: an updated systematic review and meta-analysis. *Cognitive Behaviour Therapy*. Routledge.

The ImpleMentAll project received funding from the European Union's Horizon 2020 research and innovation programme, under the Grant Agreement No. 733025

The information in this document is provided as is and no guarantee or warranty is given that the information is fit for any particular purpose. The user thereof uses the information at its sole risk and liability. This content reflects only the author's view and the European Commission is not responsible for any use that may be made of the information it contains.

https://doi.org/10.1080/16506073.2017.1401115

Cuijpers, P., Noma, H., Karyotaki, E., Cipriani, A., & Furukawa, T. A. (2019). Effectiveness and Acceptability of Cognitive Behavior Therapy Delivery Formats in Adults with Depression: A Network Meta-analysis. *JAMA Psychiatry*. https://doi.org/10.1001/jamapsychiatry.2019.0268

[iv] Etzelmueller, A., Vis, C., Karyotaki, E., Baumeister, H., Titov, N., Berking, M., … Ebert, D. D. (2020, August 31). Effects of Internet-Based Cognitive Behavioral Therapy in Routine Care for Adults in Treatment for Depression and Anxiety: Systematic Review and Meta-Analysis. *Journal of Medical Internet Research*. NLM (Medline). https://doi.org/10.2196/18100

[v] Romijn, G., Batelaan, N., Kok, R., Koning, J., van Balkom, A., Titov, N., & Riper, H. (2019). Internet-Delivered Cognitive Behavioral Therapy for Anxiety Disorders in Open Community Versus Clinical Service Recruitment: Meta-Analysis. *Journal of Medical Internet Research*, *21*(4). https://doi.org/10.2196/11706

[vi] Andersson, G., Titov, N., Dear, B. F., Rozental, A., & Carlbring, P. (2019). Internet-delivered psychological treatments: from innovation to implementation. World Psychiatry, 18(1), 20–28.

[vii] Vis, C., Mol, M., Kleiboer, A. M., Bührmann, L., Finch, T. L., Smit, J. H., & Riper, H. (2018). Improving Implementation of eMental Health for Mood Disorders in Routine Practice: Systematic Review of Barriers and Facilitating Factors. JMIR Mental Health, 5(1), e20–18.

The ImpleMentAll project received funding from the European Union's Horizon 2020 research and innovation programme, under the Grant Agreement No. 733025
The information in this document is provided as is and no guarantee or warranty is given that the information is fit for any particular purpose. The user thereof uses the information at its sole risk and liability. This content reflects only the author's view and the European Commission is not responsible for any use that may be made of the information it contains.
